# Multivariate Curve Resolution Methodology Applied to the ATR-FTIR Data for Adulteration Assessment of Virgin Coconut Oil

**DOI:** 10.3390/molecules28124661

**Published:** 2023-06-09

**Authors:** Michele De Luca, Giuseppina Ioele, Fedora Grande, Maria Antonietta Occhiuzzi, Martina Chieffallo, Antonio Garofalo, Gaetano Ragno

**Affiliations:** Department of Pharmacy, Health and Nutritional Science, University of Calabria, 87036 Rende, Italy; giuseppina.ioele@unical.it (G.I.); fedora.grande@unical.it (F.G.); mariaantonietta.occhiuzzi@unical.it (M.A.O.); antonio.garofalo@unical.it (A.G.); gaetano.ragno@unical.it (G.R.)

**Keywords:** virgin coconut oil, food analysis, infrared spectroscopy, multivariate analysis, calibration model, variable selection, genetic algorithm, control chart

## Abstract

Virgin coconut oil (VCO) is a functional food with important health benefits. Its economic interest encourages fraudsters to deliberately adulterate VCO with cheap and low-quality vegetable oils for financial gain, causing health and safety problems for consumers. In this context, there is an urgent need for rapid, accurate, and precise analytical techniques to detect VCO adulteration. In this study, the use of Fourier transform infrared (FTIR) spectroscopy combined with multivariate curve resolution–alternating least squares (MCR-ALS) methodology was evaluated to verify the purity or adulteration of VCO with reference to low-cost commercial oils such as sunflower (SO), maize (MO) and peanut (PO) oils. A two-step analytical procedure was developed, where an initial control chart approach was designed to assess the purity of oil samples using the MCR-ALS score values calculated on a data set of pure and adulterated oils. The pre-treatment of the spectral data by derivatization with the Savitzky–Golay algorithm allowed to obtain the classification limits able to distinguish the pure samples with 100% of correct classifications in the external validation. In the next step, three calibration models were developed using MCR-ALS with correlation constraints for analysis of adulterated coconut oil samples in order to assess the blend composition. Different data pre-treatment strategies were tested to best extract the information contained in the sample fingerprints. The best results were achieved by derivative and standard normal variate procedures obtaining RMSEP and RE% values in the ranges of 1.79–2.66 and 6.48–8.35%, respectively. The models were optimized using a genetic algorithm (GA) to select the most important variables and the final models in the external validations gave satisfactory results in quantifying adulterants, with absolute errors and RMSEP of less than 4.6% and 1.470, respectively.

## 1. Introduction

*Cocos nucifera* L. is a palm belonging to the Arecaceae family, native to the Eastern tropical regions, mainly cultivated in Asia, Central and South America, and Africa [1]. Coconut oil can be extracted through methods that can be divided into wet and dry processes [2,3,4]. Virgin coconut oil (VCO) is an edible oil obtained from the extraction of mature coconut kernels and produced by a variety of extraction processes, hot or cold, or by fermentation, centrifugation, and extraction from dried coconuts [5]. The different procedures have the aim of minimizing the degradation processes of the nutritional components and ensuring a low level of acidity. It does not undergo any chemical treatment to produce refined/bleached/deodorized oil [2]. The main VCO components are saturated fatty acids, which constitutes about 94% of the weight, with about 62% of medium-chain fatty acids. Among these last, lauric acid is most abundant with a percentage range of 46–48% by weight [6]. VCO has assumed a significant role in recent years thanks to the discovery of some biological and healthy properties such as antioxidant, anti-inflammatory, antihyperlipidemic, and antibacterial activity due to some substances such as phenols and tocopherols [7]. VCO and coconut oil are traditionally used as beauty products, to promote hair growth, and hydrate the skin, they are also used for minor illnesses such as diarrhea and skin inflammation [8,9,10].

The commercial price of VCO is about ten times higher than that of common vegetable oils, making it a potential target for adulteration. This is undoubtedly a source of concern for both buyers and the food industry [11]. The most common adulteration of high-cost vegetable oil consists of a blending process with cheaper edible or non-edible oils [11,12,13]. The labeling of edible oils, such as the identification and quantification of adulterations, has increased the attention of many researchers and centers specialized in food matrices. Therefore, with the aim of avoiding fraud, advanced and reliable PAT (process analytical technology) strategies for the certification of pure coconut oil and the detection of various adulterants have been studied. Indeed, several analytical procedures have been developed, such as gas chromatography [14], high-performance liquid chromatography [15], electronic nose [16], differential scanning calorimetry [17], and nuclear magnetic resonance spectroscopy [18]. Undoubtedly, the mentioned approaches are sensitive and accurate but often require high instrumentation costs, complex and time-consuming sample pre-treatment, as well as solvent use and sample destruction.

The coupling between vibrational spectroscopic techniques and chemometric tools able to extract and handle information from complex chemical systems has become a trending topic in the case of characterization and authentication of food matrices. Analytical investigations on edible oils are certainly a much-discussed topic in this sector. The different vibrational approach is generally simple to carry out, low expensive in terms of time and money, and usually requires minimum or no sample pre-treatment [19,20,21]. Indeed, near-infrared (NIR), mid-infrared (MIR), and Raman spectroscopy are characterized by rapid spectral acquisition without the sample being destroyed and provide useful qualitative–quantitative information about oil samples [2,22]. This information can be processed by different chemometric approaches. Multivariate elaborations can be carried out by unsupervised pattern recognition applying principal component analysis (PCA) or hierarchical cluster analysis (HCA), or supervised classification. Partial least squares discriminant analysis (PLS-DA), SIMCA, and linear discriminant analysis (LDA) methodologies have been successfully used to discriminate purely from adulterated coconut oil samples [5,21,23,24]. PLS and PCR (principal component regression) algorithms have been also used to build calibration models to determine oil adulteration amount [25,26,27]. All the previous examples are able to detect and, in many cases, determine the number of adulterants with satisfactory results, however, they allow for poor interpretation of the spectral information, and have some difficulty in distinguishing adulterants. A valid alternative is to exploit methodologies based on the multivariate curve resolution, alternating least squares (MCR-ALS), where the vibrational spectroscopic data can be processed by simultaneously acquiring information about the concentration and spectral features of the samples, detecting the pure spectral profiles of the adulterants in the oil mixtures [20,21].

Coconut oil adulterations have been investigated by using different vibrational and chemometric tools. FTIR data were used to detect adulteration of VCO with different low-cost oils using multivariate approaches [13]. PCA, PCR, and LDA algorithms were applied to infrared data in order to detect the addition of fried coconut, paraffin, mustard, and palm oils [24,28,29]. Raman spectroscopy was coupled with MCR-ALS modeling to assay adding of sunflower, canola oils, and Vaseline [30].

**Figure 1:**
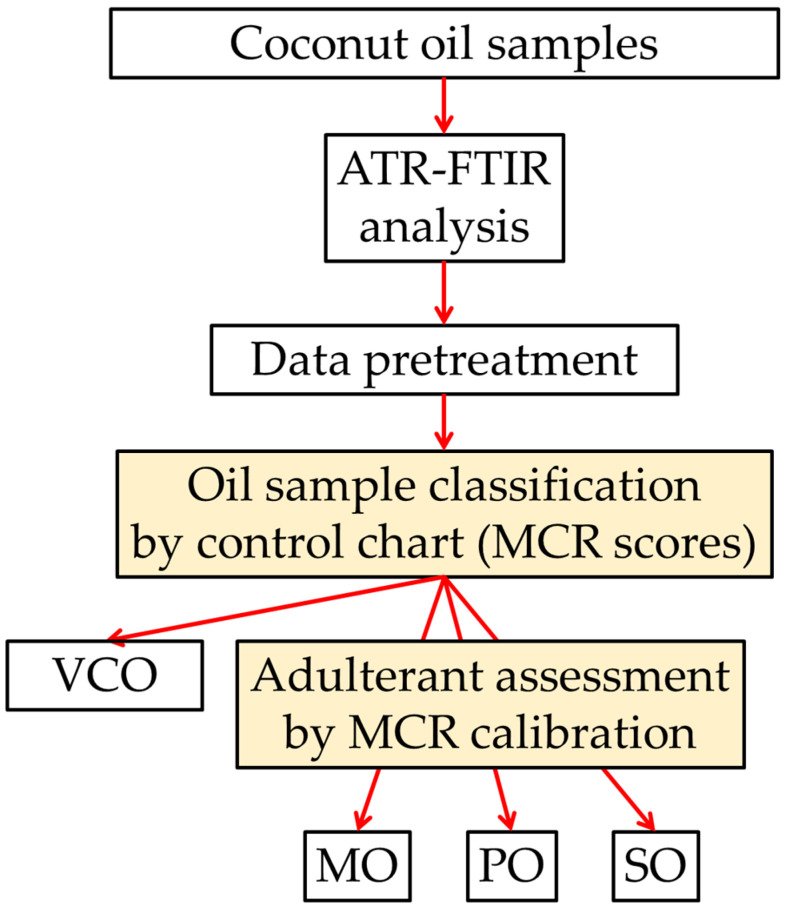
Scheme of the flow PAT tool for adulteration evaluation of virgin coconut oil samples by MCR-ALS and FTIR spectra.

In this work, multivariate curve resolution modeling was applied to FTIR data to define a flow PAT tool for the detection and quantification of VCO adulteration by three low commercial value oils, sunflower (SO), corn (or maize, MO), and peanut (PO) oils (Figure 1). In the first step, the multivariate resolution was used to discriminate pure from blended VCO and, in the second step, to quantify the addition of adulterants by the evaluation of the oil fingerprints. The predictive performance of the models was evaluated with respect to different types of data pre-treatment and optimization procedures. To the best of our knowledge, this is the first time that MCR methodologies are being exploited in FTIR data processing by evaluating different data pre-processing procedures and selecting variables on the models’ ability to analyze virgin coconut oil.

## 2. Results and Discussion

### 2.1. Comparison among FTIR Spectra of Pure Virgin Coconut Oil and Adulterants

**Figure 2:**
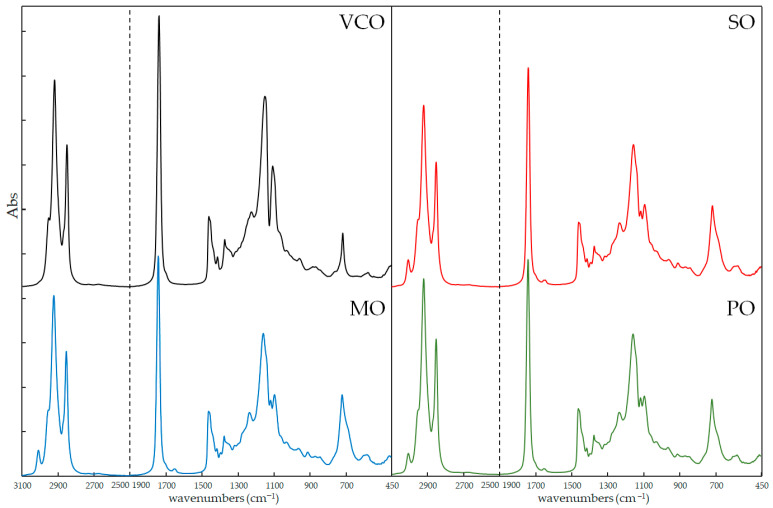
FTIR spectra for each vegetable oil: virgin coconut oil (VCO); sunflower oil (SO); maize oil (MO); peanut oil (PO).

Figure 2 shows the FTIR spectra of pure VCO and the adulterants SO, MO, and PO in the most informative wave regions (3100–2500 cm^−1^ and 1900–450 cm^−1^). The characteristic peaks of bond vibrations of VCO could be attributed to the functional groups of its fatty acids: 2954, 2922, and 2853 cm^−1^ due to stretching of -C-H (-CH_2_ and -CH_3_); 1741 cm^−1^ for stretching of ester group -C=O; 1466 and 1417 cm^−1^ for bending of -C-H (-CH_2_ and -CH_3_) and =C-H (cis); 1228, 1155 and 1111 cm^−1^ for stretching and bending of -C-O and -C-O-CH_2_; 721 cm^−1^ for bending of -(CH_2_)n-.

**Figure 3:**
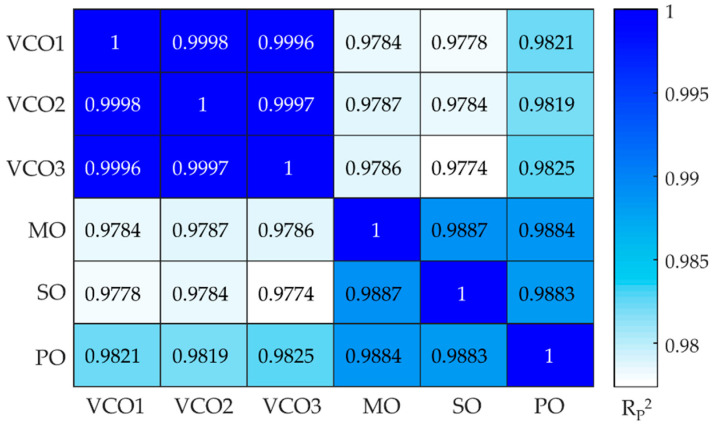
Pearson correlation coefficient (Rp^2^) calculated for all vegetable oils.

In the informative regions, the adulterants showed some differences in the spectra due to their different compositions, the unsaturated long chain with -C-H stretching frequency appeared at 3007 cm^−1^, while the ester bond of C-O stretching was evident around 1118 and 1097 cm^−1^. Except for these few differences, the overlap of the oil spectra was substantial and even more among the adulterants, as confirmed by the calculation of the Pearson correlation coefficient (R_P_^2^) as plotted in Figure 3. The correlation coefficients were higher than 0.9996 when the comparison was among different brands of VCO, while the values decreased to values below 0.9825 for VCOn versus adulterants. Among the adulterant oils, the R_P_^2^ values were in the range 0.9883–0.9887 [31].

Qualitative–quantitative evaluation by classical spectroscopy using discrete wave variable information appeared unsuitable due to the large overlap of the spectral curves. Therefore, it appeared necessary to perform a multivariate study of the data to interpret the data matrices, taking into account all information from the FTIR fingerprints of the samples.

### 2.2. Adulteration Detection by Multivariate Resolution of Pure and Blended VCO Samples

First of all, when assessing VCO adulteration, a prior distinction should be made between pure and adulterated oil samples. For this purpose, the MCR-ALS algorithm was used, taking into account the ability of multivariate curve resolution to distinguish the contributions of the different components in the spectral data obtained from complex mixtures. A column-wise augmented matrix **D_aug,cl_** (**D_aug,cl_** = [**D_t_**;**D_cl_**] in Matlab notation) for MCR analysis consisting of two subsets was arranged. The first subset **D_t_** contained the samples used as a training set with pure coconut oil samples belonging to all considered brands (9 VCO samples × 2025 wave variables) and the samples blended with the adulterants maize, peanut, and sunflower oils (70 CMO + 70 CPO + 70 CSO × 2025). The second matrix **D_cl_** (6 VCO + 20 CMO + 20 CPO + 20 CSO) contained pure VCO and adulterated samples to be subjected to classification.

The MCR-ALS algorithm with non-negativity constraints applied in both concentration and spectral optimization decomposed matrix **D_aug,cl_** providing scores related to the composition of each sample in the matrix, and these values were used to implement the control charts [30]. Sample classification by means of the control charts was carried out by considering the mean and the standard deviation of the score values calculated for the unadulterated reference samples. The classification limits were determined by adding and removing two times the standard deviation from the mean value of the scores to generate the minimum and maximum limits, respectively. Control and unadulterated samples had to be within the limits; consequently, samples appearing outside these limits were defined as adulterated.

**Figure 4:**
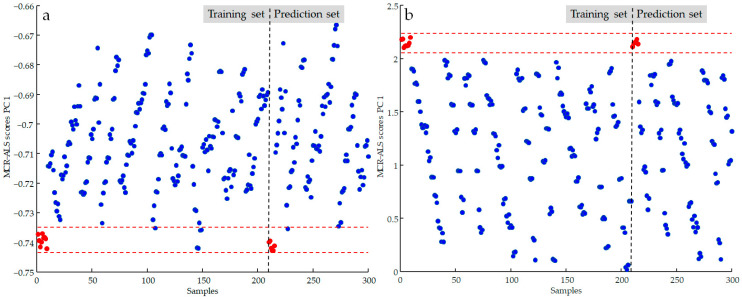
Control chart model developed by considering the scores values calculated using MCR-ALS algorithm (black dotted lines for separation of training and validation samples; red dotted lines represent the limits for sample classification): (**a**) control chart produced using FTIR original data; (**b**) control chart produced using data subdued to derivative pre-treatment.

Figure 4 shows the control chart built for the samples described in **D_aug,cl_**. The report graph is divided into training and prediction sections, and the dashed red lines delimit pure samples from adulterated ones for both sections. In this first processing, all pure samples analyzed in the prediction set were correctly classified, as all score values were in the range −0.7349 and −0.7434. However, it is evident that some of the values calculated for the adulterated samples place them in the selected range by misclassifying them. When the adulterants were mixed at lower concentrations, the iterative MCR process was unable to distinguish the components of the mixture without being able to identify the adulteration.

In an effort to make the information contained in spectral signals more available and help the algorithm process the data, the effect of certain pre-pretreatment procedures on classification performance was tested. Derivatization by the Savitzky–Golay algorithm, standard normal variate (SNV), and multiple scatter correction (MSC) were applied to the FTIR recorded data [24,32]. The classification of the oil samples was repeated by using the transformed **D_aug,cl_** matrix after all data pretreatments. A significant improvement in the useful variance with the derivative transformation of the spectral signals. Different operative conditions were tested in applying the derivative calculation and the best results were reached with the following parameters: 1st order, number of smoothing points 7, and polynomial order 2. In Figure 4b, it is evident how the control chart range between the values 2.05297 and 2.23724 of the MCR scores calculated with the derivate data was able to correctly classify all the samples of both training and prediction sets. However, MCR processing using derivative-transformed spectral data required appropriate data handling: when using absorbance spectral data, the non-negativity constraint is usually applied to both concentration and spectral profiles; in contrast, when using derivative data, the non-negativity constraint is only imposed on the concentration values.

These results showed that the spectral data processing was able to distinguish pure from adulterated samples without however, being able to distinguish the different types of adulteration. Therefore, it was necessary to proceed with the quantitative assessment of adulteration by considering the single addition of maize, peanut, and sunflower oil.

### 2.3. Quantitative Evaluation of Coconut Oil Adulterations

Three different calibration models by the MCR-ALS algorithm were built for the determination of the amount of maize, peanut, and sunflower oils added to the VCO, respectively. For this purpose, three augmented matrices (**D_aug,cal_** = [**D_cal_**;**D_p_**]) were assembled, each containing the calibration subset and the subset of the samples to be used in the model external validations. Each **D_cal_** matrix consisted of 70 samples of blended coconut oil with adulterant concentration between 5 and 50% and 5 samples of pure coconut and adulterant oils, while the **D_p_** matrix contained 22 samples of both adulterated and pure VCO.

In the first instance, the multivariate resolution of the matrices was carried out using the absorbance FTIR data in the ranges 3100–2500 cm^−1^ and 1900–450 cm^−1^. In these elaborations, the non-negativity constraint was applied to optimize the concentration and spectral profiles, and the additional calibration constraint was applied only to the concentration profiles.

**Figure 5:**
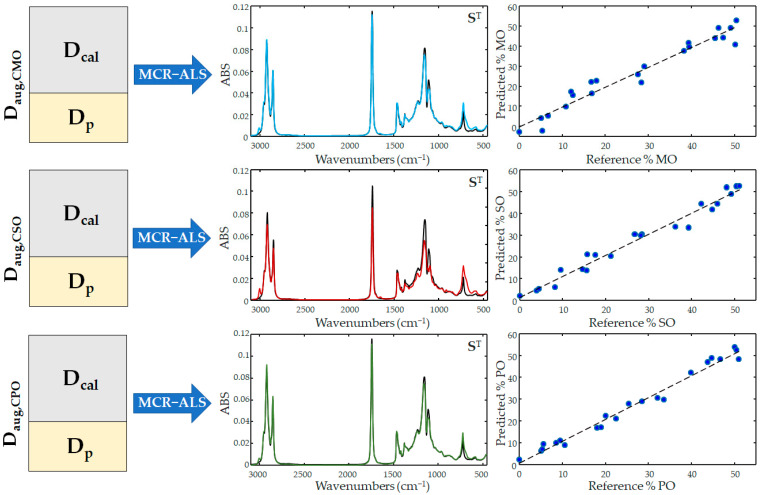
MCR calibration model on FTIR data sets; **S**^T^ matrices show the spectra obtained after multivariate resolution of the mixtures, maize (blue line), sunflower (red line), and peanut (green line) oils, respectively.

The MCR-ALS algorithm decomposed all the **D_aug,cal_** matrices into their respective **C** and **S** matrices, where it was possible to observe how the multivariate resolution was able to distinguish the composition of the oil samples, returning the pure fingerprints belonging to the VCO and to all the adulterant oils in the **S** matrices (Figure 5). Evaluating the coefficients of R_p_^2^, a very high correlation was observed between the pure spectra recorded instrumentally and the spectral profiles calculated by the MCR algorithm, as can be seen in Table 1, with correlation values above 0.92 in all cases.

Despite the good ability of the MCR algorithm to qualitatively describe the spectral contribution of each vegetable oil in the samples, the quantitative prediction performance resulted below a level that can be considered satisfactory. The adulterant detected with the lowest error was PO with a RE% of 7.95%, while for the detection of MO, this value was not less than 11.9%.

The calibrations were then repeated by replacing the augmented matrices with the data resulting from the pre-treatment of the spectral data using derivative signals, SNV, and MSC transformations. The pre-treatment strategies succeeded in all cases in improving the predictive capabilities of the calibration models: this was evident for the VCO adulteration with MO and SO, as the transformation of the data into derivatives was able to reduce the prediction error of the CMO and CSO mixtures up to 8.35% and 5.59%, respectively, while the SNV pre-treatment was the best for the CPO mixture with an error of 6.48%.

The variable selection strategy aims to select a subset of variables that can improve prediction performance and streamline the model. The Genetic Algorithm (GA) is a popular variable selection approach that uses an evolutionary selection of individuals from a larger population [33]. In the GA procedure, the sequences of genes/variables are grouped into chromosomes and used to build models. The selection of the best chromosomes is based on two steps called crossover and mutation. When evolution produces a new chromosome with better performance than the previous ones, it enters the selected population, and the worst ones are discarded. In our work, GA and PLS algorithms were used in synergy to select the best variable subset, starting from the best pre-treatment procedure [33]. The figures of merit RMSECV (root mean square error of cross-validation) and *R*^2^ calculated during the full cross-validation were considered discriminative in the selection procedures. GA selected three different subsets of matrices: for the CMO and CPO calibration models, 2 sets of 426 variables were selected for each adulteration, while for the CSO model, the GA-PLS selection gave good statistical data in terms of RMSECV and *R*^2^ with a smaller set of 284 variables. The predictive ability of the MCR-ALS models improved for all types of adulteration such that they can predict the adulteration of VCO with maize, peanut, and sunflower oils within an error range of 3.76 to 4.58%.

## 3. Materials and Methods

### 3.1. Virgin Coconut Oil Collection and Sample Arrangement

VCO of three Italian brands (VCO1, VCO2, and VCO3) and three different types of adulterant oils, maize (MO), peanut (PO), and sunflower (SO) from the same brand were purchased from the local market at Rende, Italy. Five samples from each VCO brand and adulterant oils were collected for the analysis of pure sample oils (Table 1). The blended samples were obtained by mixing each VCOn brand with one adulterant oil at a time in the range from 5 to 50% *v*/*v* with an increase of 5%, replicated three times. All oil mixtures were stored in borosilicate flasks at 4 °C, in the absence of light. Before the spectral analysis, each sample was returned to room temperature and vortexed for 1 min at 5000 rpm. Finally, 90 VCO blended samples were produced for each adulterant oil, with a total of 270 samples, as reported in Table 2. The Kennard–Stone procedure for sample partition was used to arrange calibration and testing subsets. For this purpose, 70 samples from each blended set were selected for the calibration step and 20 samples were used to validate the prediction performance of the models [34].

### 3.2. FTIR-ATR Spectra Acquisition and Treatment

FTIR spectra were acquired on a Fourier transform infrared spectrometer (Spectrum Two, Perkin Elmer, Milan, Italy) equipped with a diamond crystal cell attenuated total reflection accessory (HATR top plate fitted with a 50 mm ZnSe crystal). Oil samples were placed on the ATR surface and infrared spectra were recorded between 4000 and 450 cm^−1^. All spectra were acquired at a resolution of 4 cm^−1^ and 32 scans. After cleaning and drying the ATR element, the room air spectrum was selected as the background. The ATR plate was cleaned prior to each analysis with dry paper and wiped with hexane and ethanol, making it possible to dry the surface of the ATR. Cleanliness was verified by comparing the background spectrum with the previous ones to check the instrumental conditions and laboratory interferences from H_2_O and CO_2_. The spectral data were converted into .csv files suitable for direct import into statistical tools. The computing environment MATLAB^®^ (The Mathworks, Inc., Natick, MA, USA) and The Unscrambler X from CAMO (Computer Aided Modelling, Trondheim, Norway) were used for the handling of ATR-FTIR spectral data.

The spectral windows 4000–3100 cm^−1^ and 2500–1900 cm^−1^ were not considered due to a lack of information [24], so each sample spectrum was stored by selecting the wavenumber ranges 3100–2500 cm^−1^ and 1900–450 cm^−1^ with a data vector consisting of 2052 wave variables. According to the sample preparation and FTIR spectra acquisition, the experimental data were arranged in four data matrices, one matrix dedicated to pure oil samples with dimensions 30 × 2025 and three matrices for blended samples (70 × 2025 for each adulterant). All the matrices were divided into their respective subsets dedicated to the calibration and validation of the models, as described in the previous section. Prior to the multivariate analysis, a pre-treatment of the data was applied converting the spectral signal from reflectance to absorbance unit and the baseline correction to allow the exploitation of Beer’s law and adjust the spectral offset adjusting the data to the minimum values.

### 3.3. Chemometric Method

The vibrational spectra of food samples and their adulterants are in many cases very similar, and this is certainly the case for edible vegetable oils. Therefore, chemometric tools for instrumental data handling and spectral identification and quantification are fundamental for the development of quality control strategies. Multivariate curve resolution methodologies are characterized by their ability to process the recorded spectral data and distinguish the spectral contribution of the individual components that make up complex chemical samples, which may consist of two single substances up to highly complex natural mixtures such as food samples.

The MCR-ALS approach is based on the extraction of relevant information about the single components in a complex chemical system by means of a bilinear decomposition procedure from the experimental data matrix D (*n*,*m*), where the sample spectra (*n*) are arranged with the corresponding wavenumbers (*m*). The MCR modeling produces two smaller matrices containing information about the pure components in terms of their respective concentrations (matrix C) and spectra (matrix S); these matrices can be used for classification or quantification purposes, while the spectral profile of the components present in the complex mixtures is useful for their identification. Finally, as shown in Equation (1), where the decomposition is described mathematically, matrix E includes information not explained by the model.

**D** = **CS^T^** + **E** (1)

The MCR elaboration uses an iterative ALS (alternating least squares) algorithm, which involves the generation of a sequence of approximate solutions that change with the execution of several cycles. The ALS procedure is often unable to provide unique solutions due to intensity and/or rotational ambiguity in the elaborations, however, a series of constraints can be applied to reduce the number of responses and fit the optimized result with a chemical significance. The non-negativity constraint can only guarantee positive values for matrix C and S when applied and the correlation constraint is usually dedicated to the MCR-ALS calibration and mixture determination, even in the presence of unknown interferences. The correlation constraint is implemented in the ALS iteration, where the relationship between the reference concentrations and calibration samples is used to predict the concentration of unknown samples. Multivariate resolution was achieved by using the MCR-ALS 2.0 toolbox for Matlab^®^ [35].

In order to evaluate the prediction performance of the MCR-ALS models, external validation was made by using new samples (not used during the calibration step). The following figures of merit were calculated to describe the validation results:

Root mean squares error of prediction (*RMSEP*)

(2) $$RMSEP=\sqrt{\frac{\sum_{i=1}^{n}{\left(c_{i}-{\hat{c}}_{i}\right)}^{2}}{n}}$$

Error in predicted concentrations in% (*RE*%)

(3) $$RE\left(\%\right)=100\sqrt{\frac{\sum_{i=1}^{n}{\left(c_{i}-{\hat{c}}_{i}\right)}^{2}}{\sum_{i=1}^{n}c_{i}^{2}}}$$

Data pre-treatment procedures and variable selection were elaborated by using a regression toolbox for Matlab^®^ available on website: https://michem.unimib.it/ (accessed on 1 October 2022) [33].

## 4. Conclusions

Many of the analytical methods developed so far to quantify the adulteration of oils often require extensive use of solvents, are time-consuming, and cause the destruction of the samples. The use of FTIR spectroscopy combined with MCR-ALS analysis has proven to be a fast, clean, and non-destructive method. An analytical strategy based on two-step data processing was built, which first used the control chart method to distinguish samples of pure coconut oil from adulterated samples. The collection of the data into augmented matrices, the pre-treatment of the data, variable selection, and the use of correlation constraint, subsequently allowed the quantification of the adulterated samples using MCR calibration models ensuring that the values predicted in the concentration matrices match the effective concentration. Derivative and SNV pre-treatment approaches were very useful to improve the extraction of information from FTIR data and allowed the detection of VCO adulteration when the multivariate spectral analysis was performed. The MCR calibration models were optimized by applying a Genetic Algorithm (GA), which selected the most important variables that showed satisfactory predictive ability in assessing the addition of adulterants with errors below 4.58%. This work confirmed that ATR-FTIR spectroscopy has great potential in the control of food matrices, such as the detection of virgin coconut oil adulteration, and was particularly effective when combined with chemometric tools capable of resolving and understanding spectral signals even from complex food samples.

## Figures and Tables

**Table 1:** Figures of merit obtained for the calibration and validation of all data sets.

**Adulterant**	**Maize Oil (MO)**	**Peanut Oil (PO)**	**Sunflower Oil (SO)**
*Absorbance data*			
N. components	2	2	2
RMSEP	3.8237	2.4511	2.7890
*R* ^2^	0.9748	0.9914	0.9870
*R_p_*^2^ VCO-adulterant	0.977–0.979	0.971–0.929	0.915–0.938
RE%	11.9986	7.9505	8.6965
*Derivative data*			
N. components	2	2	2
RMSEP	2.6623	2.6754	1.7925
*R* ^2^	0.9877	0.9906	0.9944
*R_p_*^2^ VCO-adulterant	0.989–0.988	0.956–0.988	0.975–0.915
RE%	8.3540	8.6780	5.5894
*SNV data*			
N. components	2	2	2
RMSEP	2.7310	1.9991	3.1079
*R* ^2^	0.9879	0.9947	3.1079
*R_p_*^2^ VCO-adulterant	0.995–0.987	0.991–0.990	0.992–0.879
RE%	8.5699	6.4843	9.6910
*MSC data*			
N. components	2	2	2
RMSEP	4.4441	2.4318	2.8090
*R* ^2^	0.9652	0.9928	0.9867
*R_p_*^2^ VCO-adulterant	0.981–0.880	0.987–0.892	0.982–0.878
RE%	13.9452	7.8878	8.7591
Variable selection optimization procedure GA + PLS			
**Adulterant**	**Maize oil (MO)**	**Peanut oil (PO)**	**Sunflower oil (SO)**
*Data set*	*Derivative*	*SNV*	*Derivative*
*PLS factors in GA*	*3*	*3*	*2*
RMSECV	1.1747	1.1878	0.7299
*R* ^2^	0.992	0.993	0.997
N. of variables	426	426	284
Predictive performance of MCR calibration models after variable selection procedure			
N. components	2	2	2
RMSEP	1.1969	1.1937	1.4702
*R* ^2^	0.9973	0.9975	0.9962
RE%	3.7557	3.8182	4.5843

**Table 2:** Sample scheme.

Pure Sample Set	Mixture Sample Sets
VCO ^a^ brand 1 (VCO1) = 5 samples	VCO adulterated with MO 5–50%, 10 × 3 = 30 samples for each VCO brand = 90 ^c^ CMO ^b^ samples
VCO brand 2 (VCO2) = 5 samples	VCO adulterated with PO 5–50%, 30 samples for each VCO brand = 90 CPO samples
VCO brand 3 (VCO3) = 5 samples	VCO adulterated with SO 5–50%, 30 samples for each VCO brand = 90 CSO samples
MO ^a^ = 5 samples	
PO ^a^ = 5 samples	Total samples: 30 pure oil samples + 270 mixture oil samples = 300 samples
SO ^a^ = 5 samples	

^a^ VCO = virgin coconut oil; MO = maize oil; PO = peanut oil; SO = sunflower oil; ^b^ CMO = VCO + MO; CPO = VCO + PO; CSO = VCO + SO. ^c^ Adulteration procedure has been made in triplicate for each VCO brand.

## Data Availability

The data presented in this study are available on request from the corresponding author. The data are not publicly available as they contain information that could compromise the privacy of research participants.

## References

[1] Swathi S.K., Gangwal J., Pillai P.K., Rathore K., Sreejith E.J., Yadav J. (2021). A Review on Narikela (Coconut Palm). Int. Int. J. Res. Publ. Rev..

[2] Rohman A., Irnawati, Erwanto Y., Lukitaningsih E., Rafi M., Fadzilah N.A., Windarsih A., Sulaiman A., Zakaria Z. (2021). Virgin Coconut Oil: Extraction, Physicochemical Properties, Biological Activities and Its Authentication Analysis. Food Rev. Int..

[3] Bawalan D.D., Chapman K.R. (2006). Virgin Coconut Oil Production Manual for Micro- and Village-Scale Processing.

[4] Marina A.M., Che Man Y.B., Amin I. (2009). Virgin Coconut Oil: Emerging Functional Food Oil. Trends Food Sci. Technol..

[5] Ramesh S.V., Pandiselvam R., Thushara R., Manikantan M.R., Hebbar K.B., Beegum S., Mathew A.C., Neenu S., Shil S. (2020). Engineering Intervention for Production of Virgin Coconut Oil by Hot Process and Multivariate Analysis of Quality Attributes of Virgin Coconut Oil Extracted by Various Methods. J. Food Process Eng..

[6] Asiah N., Astuti R.M., Cempaka L., Setiani R. (2019). Physical and Chemical Characteristic of Virgin Coconut Oil under Mix Culture Fermentation Technique. J. Phys. Conf. Ser..

[7] Salian V., Shetty P. (2018). Coconut Oil and Virgin Coconut Oil: An Insight into Its Oral and Overall Health Benefits. J. Clin. Diagn. Res..

[8] Mansor T.S.T., Man C., Afiq A., Nurul K. (2012). Physicochemical Properties of Virgin Coconut Oil Extracted from Different Processing Methods. Int. Food Res. J..

[9] Ghani N.A.A., Channip A.-A., Chok Hwee Hwa P., Ja’afar F., Yasin H.M., Usman A. (2018). Physicochemical Properties, Antioxidant Capacities, and Metal Contents of Virgin Coconut Oil Produced by Wet and Dry Processes. Food Sci. Nutr..

[10] Marina A.M., Che Man Y.B., Nazimah S.A.H., Amin I. (2009). Chemical Properties of Virgin Coconut Oil. J. Am. Oil Chem. Soc..

[11] Priya R.B., Rashmitha R., Preetham G.S., Chandrasekar V., Mohan R.J., Sinija V.R., Pandiselvam R. (2022). Detection of Adulteration in Coconut Oil and Virgin Coconut Oil Using Advanced Analytical Techniques: A Review. Food Anal. Methods.

[12] Rohman A., Che Man Y.B. (2011). The Use of Fourier Transform Mid Infrared (FT-MIR) Spectroscopy for Detection and Quantification of Adulteration in Virgin Coconut Oil. Food Chem..

[13] Rifna E.J., Pandiselvam R., Kothakota A., Subba Rao K.V., Dwivedi M., Kumar M., Thirumdas R., Ramesh S.V. (2022). Advanced Process Analytical Tools for Identification of Adulterants in Edible Oils—A Review. Food Chem..

[14] Xu B., Li P., Ma F., Wang X., Matthäus B., Chen R., Yang Q., Zhang W., Zhang Q. (2015). Detection of Virgin Coconut Oil Adulteration with Animal Fats Using Quantitative Cholesterol by GC × GC–TOF/MS Analysis. Food Chem..

[15] Komaram A.C., Anjaneyulu E., Goswami K., Nayak R.R., Kanjilal S. (2021). Detection and Quantification of Palmolein and Palm Kernel Oil Added as Adulterant in Coconut Oil Based on Triacylglycerol Profile. J. Food Sci. Technol..

[16] Marina A.M., Man Y.B.C., Amin I. (2010). Use of the SAW Sensor Electronic Nose for Detecting the Adulteration of Virgin Coconut Oil with RBD Palm Kernel Olein. J. Am. Oil Chem. Soc..

[17] Mansor T.S.T., Man Y.B.C., Shuhaimi M. (2012). Employment of Differential Scanning Calorimetry in Detecting Lard Adulteration in Virgin Coconut Oil. J. Am. Oil Chem. Soc..

[18] Dayrit F.M., Buenafe O.E.M., Chainani E.T., de Vera I.M.S. (2008). Analysis of Monoglycerides, Diglycerides, Sterols, and Free Fatty Acids in Coconut (*Cocos nucifera* L.) Oil by 31P NMR Spectroscopy. J. Agric. Food Chem..

[19] Terouzi W., De Luca M., Bolli A., Oussama A., Patumi M., Ioele G., Ragno G. (2011). A Discriminant Method for Classification of Moroccan Olive Varieties by Using Direct FT-IR Analysis of the Mesocarp Section. Vib. Spectrosc..

[20] Castro R.C., Ribeiro D.S.M., Santos J.L.M., Páscoa R.N.M.J. (2021). Comparison of near Infrared Spectroscopy and Raman Spectroscopy for the Identification and Quantification through MCR-ALS and PLS of Peanut Oil Adulterants. Talanta.

[21] De Luca M., Ioele G., Spatari C., Caruso L., Galasso M.P., Ragno G. (2019). Evaluation of human breastmilk adulteration by combining Fourier transform infrared spectroscopy and partial least square modeling. Food Sci. Nutr..

[22] Rohman A. (2017). Infrared Spectroscopy for Quantitative Analysis and Oil Parameters of Olive Oil and Virgin Coconut Oil: A Review. Int. J. Food Prop..

[23] Neves M.D.G., Poppi R.J. (2020). Authentication and Identification of Adulterants in Virgin Coconut Oil Using ATR/FTIR in Tandem with DD-SIMCA One Class Modeling. Talanta.

[24] Amit, Jamwal R., Kumari S., Dhaulaniya A.S., Balan B., Singh D.K. (2020). Application of ATR-FTIR Spectroscopy along with Regression Modelling for the Detection of Adulteration of Virgin Coconut Oil with Paraffin Oil. LWT.

[25] Bassbasi M., De Luca M., Souhassou S., Hirri A., Berkani M., Kzaiber F., Ioele G., Ragno G., Oussama A. (2014). Determination of Milk Adulteration by Sucrose Using FT-MIR Spectroscopy and Chemometrics Methods. Agric. Res. J..

[26] Zicker M.C., Craig A.P., de Oliveira Ramiro D., Franca A.S., Labanca R.A., Ferreira A.V.M. (2016). Quantitative Analysis of Acidity Level in Virgin Coconut Oils by Fourier Transform Infrared Spectroscopy and Chemometrics. Eur. J. Lipid Sci. Technol..

[27] Amit, Jamwal R., Kumari S., Dhaulaniya A.S., Balan B., Kelly S., Cannavan A., Singh D.K. (2020). Utilizing ATR-FTIR Spectroscopy Combined with Multivariate Chemometric Modelling for the Swift Detection of Mustard Oil Adulteration in Virgin Coconut Oil. Vib. Spectrosc..

[28] Amit, Jamwal R., Kumari S., Kelly S., Cannavan A., Singh D.K. (2020). Rapid Detection of Pure Coconut Oil Adulteration with Fried Coconut Oil Using ATR-FTIR Spectroscopy Coupled with Multivariate Regression Modelling. LWT.

[29] Pandurangan M., Murugesan S., Shettu N., Gajivaradhan M. (2017). Detection of Adulteration of Coconut Oil Using Fourier Transform Infrared Spectroscopy and Chemometrics. Int. J. Stat. Appl. Math..

[30] De Géa Neves M., Poppi R.J. (2018). Monitoring of Adulteration and Purity in Coconut Oil Using Raman Spectroscopy and Multivariate Curve Resolution. Food Anal. Methods.

[31] Morozov A.N., Kochikov I.V., Novgorodskaya A.V., Sologub A.A., Fufurin I.L. (2015). Statistical Estimation of the Probability of the Correct Substance Detection in Ftir Spectroscopy. Comput. Opt..

[32] Yuan L., Meng X., Xin K., Ju Y., Zhang Y., Yin C., Hu L. (2023). A Comparative Study on Classification of Edible Vegetable Oils by Infrared, near Infrared and Fluorescence Spectroscopy Combined with Chemometrics. Spectrochim. Acta Mol. Biomol. Spectrosc..

[33] Consonni V., Baccolo G., Gosetti F., Todeschini R., Ballabio D. (2021). A MATLAB Toolbox for Multivariate Regression Coupled with Variable Selection. Chemom. Intell. Lab. Syst..

[34] Galvão R.K.H., Araujo M.C.U., José G.E., Pontes M.J.C., Silva E.C., Saldanha T.C.B. (2005). A Method for Calibration and Validation Subset Partitioning. Talanta.

[35] Jaumot J., de Juan A., Tauler R. (2015). MCR-ALS GUI 2.0: New Features and Applications. Chemom. Intell. Lab. Syst..

